# Stigma and discrimination within the Ethiopian health care settings: Views of inpatients living with human immunodeficiency virus and acquired immune deficiency syndrome

**DOI:** 10.4102/phcfm.v9i1.1314

**Published:** 2017-07-31

**Authors:** Befekadu S. Wodajo, Gloria Thupayagale-Tshweneagae, Oluwaseyi A. Akpor

**Affiliations:** 1 Department of Health Studies, College of Human Sciences, University of South Africa, South Africa

## Abstract

**Background:**

Stigma and discrimination attached to human immunodeficiency virus (HIV) and acquired immune deficiency syndrome (AIDS) have been recognised as a major obstacle to HIV prevention, treatment, care and support throughout the world. Stigma and discrimination are more devastating when they occur in health care settings where it is least expected.

**Aim:**

To explore the factors attributable to stigma and discrimination of people living with HIV in two Ethiopian rural hospitals on what they thought of health care professionals (HCPs) attending to them.

**Methods:**

A qualitative exploratory approach was used. Data collection was by means of audio-taped interview and Tesch’s content analysis approach was used. The sample size for this study was determined by saturation of data and consisted of 16 participants who were people living with HIV admitted as inpatients to the two selected hospitals in Amhara region of Ethiopia.

**Results:**

Participants’ views were grouped into: fear of contact, delay of services, substandard services, denial of care, impoliteness of health care providers, breach of confidentiality and poor patient follow-up for persons infected with HIV.

**Conclusion:**

The health care settings have been recognised as one of the contexts where HIV and AIDS-related stigmatisation and discrimination can occur. Hospital policies and institutional support should be tailored to embrace people living with HIV as the provision of institutional support is imperative in creating a good working environment and improving the commitment of HCPs so as to enable them to provide holistic care for people living with HIV and AIDS (PLWHA) without discrimination.

## Introduction

Stigma and discrimination (SAD) of people living with human immunodeficiency virus (HIV) and acquired immune deficiency syndrome (AIDS) has been a hindrance for prevention, treatment and care.^1^ HIV and AIDS-related stigmatisation and discrimination jeopardises the fight against the epidemic. Thus, it is imperative that health care policy makers and administrators give due emphasis to HIV and AIDS-related SAD reduction interventions in health care settings to address health care professionals’ (HCPs) negative attitudes and potential biases.^2^

Some SAD reduction intervention strategies have been developed and discussed across the world and they should be integral components of HIV and AIDS prevention and control in the health sector.^3^ Interventions are necessary at multiple levels to fully address and eliminate HIV and AIDS-related SAD in the health care settings. Interventions targeting and empowering HCPs and people living with HIV and AIDS (PLWHA) should be coupled with institutional policies and strategies that uphold the rights of PLWHA.^3^

The SAD reduction intervention strategy should focus on addressing its root causes among HCPs.^3^ Interventions to reduce fear of infections can include dialogue regarding the HIV and AIDS-related SAD among HCPs. Moreover, improving working environment and availing infection prevention materials that support efforts of HCPs in delivering care for PLWHA is indispensable in alleviating problems associated with fear of infection in health care settings. Training about universal precaution and giving positive reinforcement to healthy attitudes towards PLWHA are also crucial in addressing the SAD. A study undertaken in Vietnam^4^ has pointed out that there is a need to conduct HIV and AIDS-related SAD reduction interventions in health care settings so as to render quality health care for PLWHA.^4^

Studies have revealed that there are effective strategies in reducing HIV and AIDS-related SAD in the health care settings.^5^ Reducing SAD attached to HIV and AIDS can dramatically improve quality of the lives of PLWHA. It also optimises investment in HIV prevention, treatment and care. Generally, it is imperative to integrate the SAD reduction intervention strategy into the whole health care system.^6^ Few studies regarding HIV and AIDS-related SAD have been conducted in the region mainly focusing on the community perspectives. The magnitude of the HIV and AIDS-related SAD and associated factors among the HCPs working in the health care settings is unknown. Moreover, no study has been conducted regarding HIV and AIDS-related SAD reduction interventions in the region or elsewhere in the country. Hence, this study seeks to explore the factors attributable to SAD of PLWHA in two Ethiopian rural hospitals. It is envisaged that the findings from this study may assist in the development of HIV and AIDS-related SAD reduction strategies in health care settings.

## Theoretical framework

**FIGURE 1:**
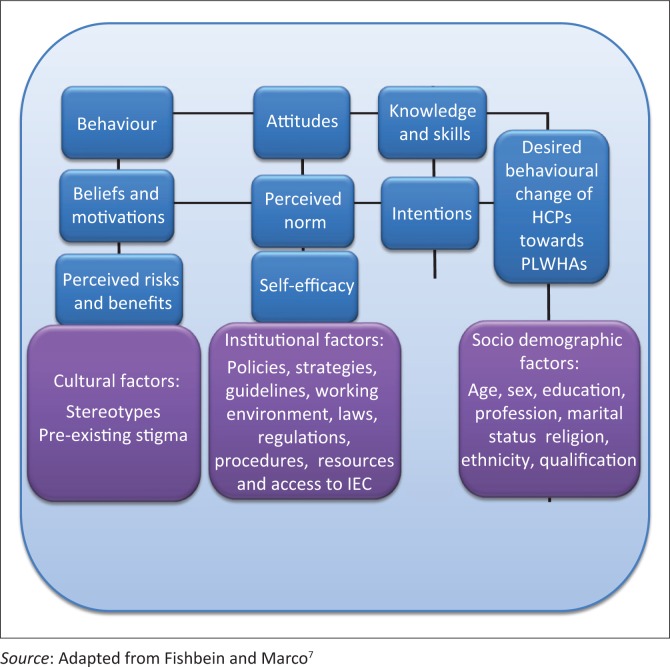
Conceptual framework for reducing HIV and AIDS-related stigma and discrimination in health care settings.

Though many theories have been applied to health-related behavioural research and designing of behavioural interventions, some argue that there are a limited number of variables that need to be considered in understanding and predicting any given behaviour. They are (1) the Health Belief Model, (2) the Theory of Reasoned Action, (3) the Social Cognitive Theory and (4) The Integrated Theoretical Model. The integrated model has been adopted for this study as illustrated in Figure 1.

Although there is empirical evidence for the role of attitudes, perceived norms and self-efficacy as determinants of intention and behaviour, support for the role of perceived risk is inconsistent. Current evidence suggests that perceived risk is the best predictor of intention and behaviour. Therefore, most behavioural theories recommend three major determinants of a person’s intentions and behaviours.^8^ They are (1) the person’s attitudes towards performing the behaviour, which depends on one’s beliefs about the positive and negative effects of performing the behaviour; (2) perceived norms, which comprise the perception that those with whom the individual interacts most closely support the person’s adoption of the behaviour and that others in the community are accomplishing the behaviour, and (3) self-efficacy, involving the person’s perception that she or he can perform the behaviour under a variety of challenging conditions. All these variables have been incorporated in an integrative model of behavioural prediction.^7^

With regard to the model, any given behaviour is most likely to occur if one has strong intentions to perform the behaviour, having the required skills as well as the necessary abilities that are required to perform the behaviour in the absence of environmental constraints that may prevent the performance of the behaviour.^7^ If there is desired intention without performance, the best intervention will be directed to either skills building or the removal of the environmental barriers. Conversely, if the strong intentions to perform a given behaviour have not been created, the model suggests three major determinants of intentions. They are attitudes towards performing the behaviour, perceived norms regarding the performance of the behaviour and one’s self-efficacy with regard to performing the specified behaviour.

## Research methods and design

### Study design

This study uses a qualitative contextual explorative approach as in-depth interviews were used.

### Setting

This study was conducted in two randomly selected referral hospitals found in two cities in Amhara region located in the north-west part of Ethiopia. In the region, there are 19 public hospitals out of which 5 are referral hospitals. The referral hospitals provide outpatient, inpatient and emergency services including all HIV and AIDS-related services, like HIV counselling and testing, and comprehensive HIV and AIDS treatment, care and support. The two referral hospitals were chosen because they had the largest HIV case load compared to the general and district hospitals.

### Study population and selection of participants

The target population for this study were PLWHA above the age of 18 years, admitted to any of the two selected referral hospitals located in north-west Ethiopia. The inclusion criteria for this study were as follows: participants would be clinically stable inpatients with AIDS-related conditions, must be residing in the study area and must have willingness to participate in the study. The sampling method was purposive and the sample size was determined by saturation of data which was achieved when 14 participants had been interviewed. A total of 14 interviews were analysed with new categories and 2 interviews analysed without new categories evolving. Referential adequacy was attained, partially fulfilling the requirement of trustworthiness.

### Data collection

Data were collected from August to September 2014. The researcher conducted and audio-taped the interviews with HIV-positive patients admitted to each referral hospital. To guide the interviewer, an interview schedule was developed to identify reduction strategy for HIV and AIDS-related SAD in health care settings. Also pre-test interviews were conducted before the actual data collection with two interviews using participants who had similar characteristics to the study population but were not included in the final data. The interviews were written and audio-recorded with the participants’ permission.

### Data analysis and trustworthiness

Interviews were transcribed verbatim and Tesch’s content analysis approach was utilised for data analysis. Data were transcribed into textual form and organised into easily retrievable sections. Then, data were coded by paragraph and further categorised and interpreted.

To ensure trustworthiness, strategies such as interpersonal relationship and trust building, triangulation of data gathering methods, peer examination, member checking and authority of the researcher, dense description structural coherence and dependability audit were employed. Participants were given a copy of their interview transcripts to review and comment on.

### Ethical consideration

Before the commencement of the study, the research proposal was approved by the Ethical Committee at UNISA (HSHDC/88/2012). After obtaining the ethical clearance, a support letter was written from UNISA Regional Office located in Addis Ababa (UNISA-ET/KA/ST/29/16-10-13) to Amhara Regional Health Bureau found in Bahir Dar. Subsequently, the proposal was submitted to the Regional Ethical Review Committee for review and it was later endorsed (HRTT/1/69/06). Finally, an official letter was written to the referral hospitals selected for this study. The managers of the two hospitals also gave their permission before the commencement of this study. Prior to the interview, each participant’s rights were explained and informed consent as well as the permission to use audio recorder was obtained. To guarantee privacy, the interviews were conducted in a private room with only the participant and the researcher present.

## Results

### Demographic profile

A total of 16 participants were interviewed (*n* = 16). As shown in Table 1, most (62.5%) of the participants were female.

**TABLE 1:** Demographic profile of the participants (*n* = 16).

Profile	*N*	%
Gender		
Female	10	62.5
Male	6	37.5
Age group (years)		
20–30	3	18.7
31–40	4	25.0
41–50	6	37.5
Above 50	3	18.7
Marital status		
Single	2	12.5
Married	10	62.5
Divorced or separated	4	25.0

### Factors related to the stigma and discrimination in the hospitals

During the interview, the inpatients inthe two hospitals described what they felt were the SAD within the hospitals under the variables explained below.

#### Fear of handling people living with HIV and AIDS

Eight of the participants in FHRH (first hospital) observed that some HCPs in the hospital use extra precautions while providing them clinical care and treatments. The patients observed that there is selective use of universal precautions that shows the existence of HIV and AIDS-related SAD in the hospital. A comment from a male patient stated:

‘I observed HCPs taking extra precautions such as wearing of gloves while taking blood pressure of the admitted HIV-positive patients.’ (P3, male, patient)

Another participant from the same hospital also said:

‘I observed HCPs showing fear of making bed of the admitted HIV-positive patients.’ (P10, female, patient)

#### Delay of services

The majority of the HIV-positive patients (*n* = 7) admitted to FHRH indicated that the care and treatment provided by the HCPs is not timely, as ordered. This results in the delay of services to the admitted patients. The extracts below attest to the participants’ beliefs about the delays. Two participants remarked:

‘I have not been getting any service for last 11 days after admission to this hospital.’ (P6, female, patient)

‘I have not been provided with the ordered treatments for the last three days.’ (P1, female, patient)

#### Substandard care

All the admitted HIV-positive patients in the two hospitals observed that the care provided to them was of low standard when compared to other admitted HIV-negative patients. A statement from a participant in FHRH was:

‘The bed linens of the HIV-positive patients in this room are not changed daily.’ (P6, female, patient)

Another participant from FHRH stated that:

‘The HCPs in this hospital seem not to have daily care plan for the HIV-positive patients admitted to the ward.’ (P12, male, patient)

#### Denial to of care

All the respondents in FHRH (*n* = 8) observed HIV-positive patients who were not allowed to be admitted to the hospital, maybe because of their HIV sero-status, as they have said. One participant from FHRH mentioned that during his stay in the hospital, he observed an HCP in the hospital refusing to admit and give care to an HIV-positive patient. His response was:

‘There was a time where one HCP refused to admit a patient because of his HIV sero-positive result.’ (P11, male, patient)

The following day, another HCP in the hospital had admitted the patient to the hospital. A senior HCP who had refused to admit the patient before came to the ward and found the patient on bed saying that:

‘I will discharge you because you were admitted to the ward without my knowledge.’ (P11, male, patient)

#### Financial discrimination

Some of the admitted HIV-positive patients in FHRH who were unable to pay for drugs indicated they are frequently requested to buy drugs for opportunistic infections. One of the participant admitted in DMRH (second hospital) said:

‘After my admission to the ward, a HCP from the ward informed me to buy the prescribed drugs as soon as possible, but I did not have money. That was a big challenge to me.’ (P5, male, patient)

Another participant also stated:

‘Just after my admission to the ward, I was examined for sputum and I was found to be positive for tuberculosis. The HCP in my ward informed me to buy the anti-tuberculosis drug, but I was unable to buy. I did not know what to do at that time.’ (P7, female, patient)

#### Impoliteness of health care providers

Half (*n* = 8) of the respondents in FHRH observed that some of the HCPs in the hospital are impolite while providing care to HIV-positive patients. A participant remarked:

Some of the HCPs in the hospital are discourteous and thus they do not show respect to the admitted HIV-positive patients.’ (P16, female, patient)

One participant stated:

‘The HPC that attended to me when I was admitted was not too friendly, she could not even make eye contact with me, and she used gloves for everything even when she took my blood pressure.’ (P7, male, patient)

#### Breach of confidentiality

Some of the participants in FHRH mentioned that some of the HCPs in the hospital do not protect the confidentiality of the admitted HIV-positive patients. One participant remarked:

‘I always hear HCPs talking about sero-status of PLWHA admitted to the ward to other HCPs openly so that even those not aligned to the care of patients will hear.’ (P14, female, patient)

Another participant stated:

‘I fear giving full information about my past history to the HCPs in my ward because I do not think the HCPs keep the secret.’ (P3, male, patient)

#### Poor follow-up of the patients

Twelve participants observed that the HCPs working in the hospitals do not provide care and treatment as expected. In addition, the patients have indicated that the HCPs in the hospitals do not regularly follow up on the condition of the admitted HIV-positive patients. A male patient in the DMRH said:

‘Some HCPs in this hospital do not provide care to the admitted HIV-positive patients.’ (P7, male, patient)

Another participant remarked:

‘I observed that HCPs assigned to my ward are not critically following the status of the admitted HIV patients. They do not ask whether or not the condition of the patients is improved.’ (P5, male, patient)

### Participants recommendations

In order to address the aforementioned discriminatory practices in the hospitals, the participants made a number of recommendations, which are outlined below.

#### Provision of training

Many of the respondents (*n* = 8) in FHRH indicated that training of the HCPs may help them provide appropriate treatments and care to HIV-positive patients admitted to the hospital. The patients suggested that training for the HCPs should not only target improvement in knowledge but also the focus on attitudes and behavioural change of the HCPs. They added that the change in attitudes and behaviour would enable the HCPs to approach the HIV-positive patients in a welcoming manner and to also deliver standard treatment and care.

#### Availing drugs for opportunistic infections

Most of the respondents (*n* = 8) in the FHRH indicated that all HIV-positive patients admitted to the hospitals who are unable to pay should get exempted services, especially drugs use for opportunistic infections.

#### Good standard of care

The majority of the respondents (*n* = 7) in FHRH recommended that the hospital should establish and implement standardised care for all patients admitted to hospital regardless of their HIV sero-status. They suggested that good standard of care can reduce the delay of services, impoliteness of the HCPs and breach of confidentiality among the HCPs. They also added that this will improve patients’ adherence to treatments and follow-ups, inspire the HCPs and improve their commitments to deliver holistic care to all the patients regardless of their HIV sero-status.

## Discussion

Findings from this study demonstrate that elimination of SAD is far from being realised as HCPs are also active participants in promoting SAD among PLWHA. Similarly, Neuman et al.^9^ affirm these findings that the quality of care is compromised for PLWHA because of stigma. More than three decades after the advent of HIV and AIDS, it still appears as if basic knowledge on transmission of HIV is elusive as participants in the study reported that it appeared HCPs feared casual contact. Previous researchers such as Saki et al.^10^ and Famoroti et al.^11^ attested to the fact that HCPs have basic knowledge of HIV transmission but implementation of that knowledge is not always applied.

Fear of occupational exposure has also been linked to poor care given to PLWHA.^11^ This study further verified quality issues such as delay of service and substandard care. These findings are supported by the findings of Essomba et al.^12^ in their study on the perceived experiences of patients on SAD in health care settings where PLWHA reported that they were deprived of treatment and that HCPs treated them with disdain.

Issues of confidentiality by HCPs have always been a major concern for PLWHA^11^ and this has been directly associated with lack of disclosure by patients and poor compliance with treatment.^13^ It is, therefore, legitimate to conclude that emphasis on education and a shift in policies are urgent and paramount. Practice of SAD by HCPs may have serious implication for care as PLWH and those with AIDS may keep away from health care settings and seek alternative treatment.^10^

## Conclusion and recommendations

This study provided evidence that HIV and AIDS-related SAD exits within the selected health care settings. Creation of HIV and AIDS-related institutional policies, strategies, guidelines and protocols is critical in reducing HIV and AIDS-related SAD in health care settings. It is necessary that all HCPs are exposed to timely orientations with regard to hospital policies, strategies, guidelines and protocols prior to assignment to their jobs. This will assist HCPs to deliver quality care and treatment based on the policies, strategies, guidelines and protocols. This will help to reduce discriminatory practices among the HCPs. However, the establishment of the policies, strategies, guidelines and protocols in the health care settings is meaningless unless it is implemented accordingly.^14^

The health care settings also need to enact hospital policies and strategies that protect the safety of the patients and HCPs. The policies and strategies need to be developed using participatory approaches, clearly communicated and regularly monitored after the implementation. Likewise, health care settings should address issues related to HIV- positive HCPs by setting appropriate hospital strategies and standard procedures. Provision of institutional support is imperative in creating a good working environment and improving the commitment of HCPs so as to enable them to provide holistic care for PLWHA without discrimination. In addition, good leadership and effective management skill of hospital managers are essential in inspiring the HCPs to provide good quality of care for PLWHA.
